# SNP imputation bias reduces effect size determination

**DOI:** 10.3389/fgene.2015.00030

**Published:** 2015-02-09

**Authors:** Pouya Khankhanian, Lennox Din, Stacy J. Caillier, Pierre-Antoine Gourraud, Sergio E. Baranzini

**Affiliations:** Department of Neurology, University of California San FranciscoSan Francisco, CA, USA

**Keywords:** genomics, genome-wide association study, SNP imputation, haplotype estimation

## Abstract

Imputation is a commonly used technique that exploits linkage disequilibrium to infer missing genotypes in genetic datasets, using a well-characterized reference population. While there is agreement that the reference population has to match the ethnicity of the query dataset, it is common practice to use the same reference to impute genotypes for a wide variety of phenotypes. We hypothesized that using a reference composed of samples with a different phenotype than the query dataset would introduce imputation bias. To test this hypothesis we used GWAS datasets from Amyotrophic Lateral Sclerosis (ALS), Parkinson Disease (PD), and Crohn's Disease (CD). First, we masked and then performed imputation of 100 disease-associated markers and 100 non-associated markers from each study. Two references for imputation were used in parallel: one consisting of healthy controls and another consisting of patients with the same disease. We assessed the discordance (imprecision) and bias (inaccuracy) of imputation by comparing predicted genotypes to those assayed by SNP-chip. We also assessed the bias on the observed effect size when the predicted genotypes were used in a GWAS study. When healthy controls were used as reference for imputation, a significant bias was observed, particularly in the disease-associated markers. Using cases as reference significantly attenuated this bias. For nearly all markers, the direction of the bias favored the non-risk allele. In GWAS studies of the three diseases (with healthy reference controls from the 1000 genomes as reference), the mean OR for disease-associated markers obtained by imputation was lower than that obtained using original assayed genotypes. We found that the bias is inherent to imputation as using different methods did not alter the results. In conclusion, imputation is a powerful method to predict genotypes and estimate genetic risk for GWAS. However, a careful choice of reference population is needed to minimize biases inherent to this approach.

## Introduction

In genome-wide association studies (GWAS), SNP data are used to find genetic loci associated with various traits, particularly common diseases. Due to the large number of tests performed (more than 2.5 million for the latest chips), correction for multiple hypothesis testing is necessary to avoid type I errors. However, after correction, there is typically not enough power to detect small effects (OR < 1.5), even with sample sizes exceeding 1000 cases and 1000 controls. A usual approach used in order to increase *n*, consists in merging datasets from two or more sources in a meta-analysis. However, if datasets were generated using different genotyping platforms (a likely scenario), a small minority of the total number of markers will be represented in both. In this case, non-overlapping genotypes are imputed using one or more reference populations (Guan and Stephens, 2008; Huang et al., 2009; Nothnagel et al., 2009; Zheng et al., 2011). Imputation methods are used to infer missing or untyped SNP genotypes based on known information (e.g., linkage disequilibrium between missing or untyped SNPs and their flanking typed SNPs) and can provide partial solutions for recovering missing or untyped genotype data (Stephens et al., 2001; Greenspan and Geiger, 2004; Browning and Browning, 2007). Several imputation methods using various statistical models such as the haplotype-clustering algorithm (Scheet and Stephens, 2006), the hidden Markov model (HMM) (Marchini et al., 2007), and the Markov Chain model (Li et al., 2006), have been proposed. Imputed genotypes, generated with these methods, have been used, successfully, to improve power in association analyses (Scott et al., 2007; Servin and Stephens, 2007; Sandhu et al., 2008; Sanna et al., 2008), to facilitate meta-analyses (Patsopoulos et al., 2011), and to replicate significant findings in follow-up studies (Willer et al., 2008).

Specifically, SNP imputation uses knowledge about haplotype structure in a densely genotyped population [often healthy controls from the HapMap International Consortium (2005), Jostins et al. (2011) or as more recently proposed, the 1000 genomes Project (Abecasis et al., 2010)] to infer unknown genotypes in the query population. Though popular SNP imputation algorithms vary in their details, they are based on the same general principle. To impute genotypes for a given individual, that individual's genotype is compared to the genotypes in the reference population (e.g., the 1000 genomes data). At each genomic region, a set of individuals from the reference population which closely matches the individual is chosen. The individual's genotype is assigned (i.e., “imputed”) based on a consensus using matching individuals from the reference panel. Thus, the imputed genotype is derived from and highly dependent on the genotypes in the reference population. This strategy can increase the power of a GWAS, enable replication of findings from different array types, and allow testing on a large number of SNPs to reveal the fine structure of an association peak (Marchini et al., 2007; Sanna et al., 2008; Willer et al., 2008; Zeggini et al., 2008; Becker et al., 2009; De Jager et al., 2009; Hao et al., 2009).

Here we analyze publically available data on three complex diseases and reveal a bias in SNP imputation that may confound this approach. Our results suggest that when solely healthy controls are used as reference for imputation, “risk” variants in the target population are more likely to be mistakenly imputed as “non-risk” alleles resulting in a deflation of the effect size in a GWAS.

## Results

To test the hypothesis that imputation introduces a systematic bias that ultimately results in a deflation of the effect size, we used three publically available datasets in Amyotrophic Lateral Sclerosis (ALS), Crohn's Disease (CD), and Parkinson's Disease (PD). For each dataset, the following steps were performed. We randomly split the dataset into two groups of equal age, gender, and case/control distribution. The two halves were termed split A and split B. We conducted a GWAS on split A. We ranked all SNPs by GWAS effect size and selected the top 100 disease associated markers (DAM) and the bottom 100 non-associated markers (NAM). We then proceeded to perform three imputations on the SNPs of interest. Each time, the SNP of interest was masked and an independent reference was used to impute the SNP. First, the cases from split A were imputed using controls from split B as reference for imputation, in order to measure the bias and error of imputation. Next, the cases from split A were imputed using cases from split B as reference for imputation, in order to see if the use of cases for imputation can improve accuracy and decrease bias. Finally, both cases and controls from the entire dataset were imputed using a commonly used publically available reference panel, in order to see if the measured effect size of a case control study is affected by using imputed genotypes vs. true genotypes.

### Discordance rate of imputation

As most imputation approaches use a healthy control population as reference, we first conducted imputation of the masked genotypes using an independent set of healthy controls as reference. When imputing integer genotypes, discordance (*D*_int_) at each SNP was defined as the percentage of samples where genotype was mistakenly inferred by imputation, and *D*_int_ = *D_M_* + *D_m_*(where *D_M_* is the percent of genotypes where imputation over-estimated the major allele by one or two copies, and *D_m_* is the percent of genotypes where imputation mistakenly over-estimated the minor allele by one or two copies). In other words, *D*_int_ is percent of genotypes which do not match imputation, composed of the cases where minor allele is mistakenly predicted *D_m_* and cases where the major allele is mistakenly predicted *D_M_*. When imputing fractional genotypes, discordance (*D*_frac_) at each SNP was defined as the average of the absolute difference between the imputed fractional genotype and the “true” genotype (coded as 0, 1, or 2; where 2 = homozygous for the major allele) across all samples, where the “true” genotype is given by SNP-chip. In general, the average *D*_int_ = 15–20% across all diseases and *D*_frac_ = 0.19 and 0.24 (Table 1). Interestingly, the lowest overall discordance rate was found in CD, the dataset with the largest sample size.

**Table 1:** **Discordance of imputation**.

Marker type^*^ (*n* SNPs)	Data^**^ (*n*)	**Mean discordance using integer genotypes [95% CI]**		**Mean discordance using fractional genotypes [95% CI]**	
		**Reference:control**	**Reference:cases**	**Reference:control**	**Reference:cases**
NAM (100)	ALS (137)	17.40% [16.30, 18.50]	17.60% [16.41, 18.80]	0.2094 [0.1970, 0.2218]	0.2108 [0.1977, 0.2240]
NAM (100)	PD (335)	15.74% [14.53, 16.96]	15.89% [14.69, 17.10]	0.1927 [0.1787, 0.2067]	0.1937 [0.1803, 0.2072]
NAM (100)	CD (406)	15.73% [14.64, 16.81]	16.13% [15.02, 17.26]	0.1935 [0.1818, 0.2051]	0.1978 [0.1857, 0.2099]
DAM (100)	ALS (137)	19.65% [18.50, 20.80]	18.04% [16.90, 19.18]	0.2311 [0.2188, 0.243]	0.2156 [0.2031, 0.2280]
DAM (100)	PD (335)	19.12% [17.84, 20.41]	18.22% [16.83, 19.61]	0.2274 [0.2128, 0.2421]	0.218 [0.2022, 0.2337]
DAM (100)	CD (406)	15.77% [14.68, 16.87]	15.45% [14.34, 16.56]	0.1918 [0.1795, 0.2040]	0.1883 [0.1759, 0.2007]

* *NAM, non-associated markers, DAM, disease-associated markers*.

** *Cases were imputed from ALS, Amyotrophic Lateral Sclerosis; PD, Parkinson's Disease; CD, Crohn's Disease*.

We observed that the imputation discordance rate (either *D*_int_ or *D*_frac_) was significantly higher for DAM than for NAM. For example, in the ALS dataset, *D*_int_ = 19.65% ± 1.16 (mean ± 2 × standard error) for DAM and *D*_int_ = 17.4% ± 1.11 for NAM, a statistically significant difference (*p* = 0.005) (Table 1). Similarly, imputation of PD samples was significantly less accurate (*p* = 0.0002) for the 100 DAM (*D*_int_ = 19.12% ± 1.3) than for 100 NAM (*D*_int_ = 15.74% ± 1.23). We found no difference in *D*_int_ between DAM and NAM in the CD dataset (*p* = 0.95). A similar pattern was found when using fractional genotypes (*D*_frac_) (Table 1).

When independent cases were used as a reference for imputation (instead of controls), a significant reduction in *D*_int_ or *D*_frac_ was observed. For example, in the ALS dataset, the average discordance at DAM (*D*_int_ = 18.04% ± 1.15) using cases as reference was significantly lower (matched pairs *t*-test, *p* = 10^−4^) than the average discordance at the same SNPs using controls as reference (*D*_int_ = 19.65% ± 1.16), suggesting that more accurate imputation is obtained when matched cases are used as reference for imputation. The average *D*_int_ in the ALS dataset using independent ALS cases as a reference was 18.04% (± 1.15) for DAM and 17.6% (± 1.2) for NAM, a non-significant difference (*p* = 0.6) (Table 1).

Similar results were obtained in the PD dataset, in which the average discordance at DAM using controls as reference (*D*_int_ = 19.12% ± 1.3) was significantly higher than that observed when matched cases were used instead (*D*_int_ = 18.22% ± 1.4) (matched pairs *t*-test, *p* = 0.002). Although using independent cases as a reference reduces the discordance in imputation, a significant drop in imputation accuracy was still observed in the PD dataset for DAM (*D*_int_ = 18.22% ± 1.4) compared with NAM (*D*_int_ = 15.89% ± 1.21) (*p* = 0.01) (Table 1). We observed similar results using fractional imputation metrics.

In summary, the average total discordance rate of imputation on each data set ranged from 15 to 20% and SNPs with large effect sizes were significantly more discordant than SNPs with small effect sizes (up to 3.5%) in two of three datasets (PD and ALS). This discordance was attenuated when cases, instead of controls, were used as reference for imputation.

### SNP imputation bias

In order to determine whether the discordance observed was random or systematic, we looked at bias. For integer imputation, we defined bias (*B*) as the difference in imputation discordance when predicting the major (*M*) and minor (*m*) alleles, *B*_int_ = *D_M_* − *D_m_*. When using fractional imputation, bias (*B*_frac_) is defined at each SNP as the average of the signed difference between the imputed fractional genotype and the true genotype (coded as 0, 1, or 2; where 2 is homozygous for the major allele) across all samples. According to these definitions, a positive bias means imputation favors the major allele, and a negative bias means imputation favors the minor allele.

Before discussing the difference between using cases or controls as a reference, or between DAM and NAM, it is important to note that imputation is inherently biased toward the minor allele. For integer genotypes, when using controls as a reference to impute NAM (markers not associated with disease), there is a small significant bias toward the major allele of 1–2% in all three datasets (ALS *p* = 0.0067; PD *p* < 10^−6^; and CD *p* < 10^−7^), Table 2. This effect was reduced by using fractional imputation (Table 2). This inherent bias is the reason that we defined bias in terms of major or minor allele, and the reason that we split the DAM (disease markers) into two groups for further analysis: markers where the major allele is the susceptibility (“risk”) allele, and markers where the minor allele is the risk allele.

**Table 2:** **Bias of imputation**.

Marker type^*^ (*n* SNPs)	**Risk allele**	Dataset^**^ (*n* samples)	Mean Bias using integer genotypes^***^ [95% CI]		Mean Bias using fractional genotypes^***^ [95% CI]	
			**Reference:controls**	**Reference:cases**	**Reference:controls**	**Reference:cases**
NAM (100)	–	ALS (137)	1.02% [0.29, 1.75]	1.64% [0.77, 2.51]	−0.0019 [−0.009, 0.0053]	0.0071 [−0.002, 0.0161]
NAM (100)	–	PD (335)	1.67% [1.04, 2.30]	1.93% [1.17, 2.68]	0.0005 [−0.0056, 0.0067]	0.0039 [−0.0035, 0.0113]
NAM (100)	–	CD (406)	1.55% [1.05, 2.05]	1.94% [1.40, 2.49]	0.0017 [−0.0025, 0.0059]	0.0069 [0.0017, 0.012]
DAM (54)	Major	ALS (137)	−10.67% [−12.59, −8.75]	−4.32% [−5.57, −3.07]	−0.126 [−0.147, −0.1051]	−0.0638 [−0.0762, −0.0514]
DAM (52)	Major	PD (335)	−7.21% [−8.30, −6.12]	−2.51% [−3.81, −1.22]	−0.0934 [−0.1033, −0.0835]	−0.0394 [−0.0522, −0.0266]
DAM (47)	Major	CD (406)	−4.55% [−5.87, −3.23]	−0.90% [−1.58, −0.21]	−0.0615 [−0.0762, −0.0467]	−0.0232 [−0.0302, −0.0162]
DAM (46)	Minor	ALS (137)	12.51% [10.85, 14.18]	6.00% [3.98, 8.01]	0.1253 [0.1065, 0.1441]	0.0571 [0.0352, 0.0789]
DAM (48)	Minor	PD (335)	10.60% [9.24, 12.0]	5.98% [4.62, 7.35]	0.1018 [0.0875, 0.1161]	0.0476 [0.0328, 0.0625]
DAM (53)	Minor	CD (406)	8.97% [8.20, 9.73]	5.55% [4.37, 6.37]	0.0812 [0.0722, 0.0901]	0.047 [0.0378, 0.0561]

* *NAM, non-associated markers, DAM, disease-associated markers*.

** *Cases were imputed from ALS, Amyotrophic Lateral Sclerosis; PD, Parkinson's Disease; CD, Crohn's Disease*.

*** *Positive values indicate preference for major allele*.

When controls were used as a reference for imputation DAM, a consistent bias against the risk allele was observed for all three diseases. For example, when the major allele was the risk allele, integer imputation was biased toward the minor allele (*p* < 10^−7^ for each disease) and when the minor allele was the risk allele, imputation was biased toward the major allele (*p* < 10^−15^ for each disease). Similar results were observed when fractional imputation was used.

Interestingly, when independent cases were used as a reference to impute DAM, the bias against risk alleles remained. Similarly to what we observed using controls as a reference, when the major allele was the risk allele, imputation was significantly biased toward the minor allele (*p* < 10^−8^ in ALS; *p* = 3 × 10^−4^ in PD; and *p* = 0.012 in CD). Conversely, when the minor allele was the risk allele, imputation was significantly biased toward the major allele (*p* < 10^−6^ for each disease).

**Figure 1:**
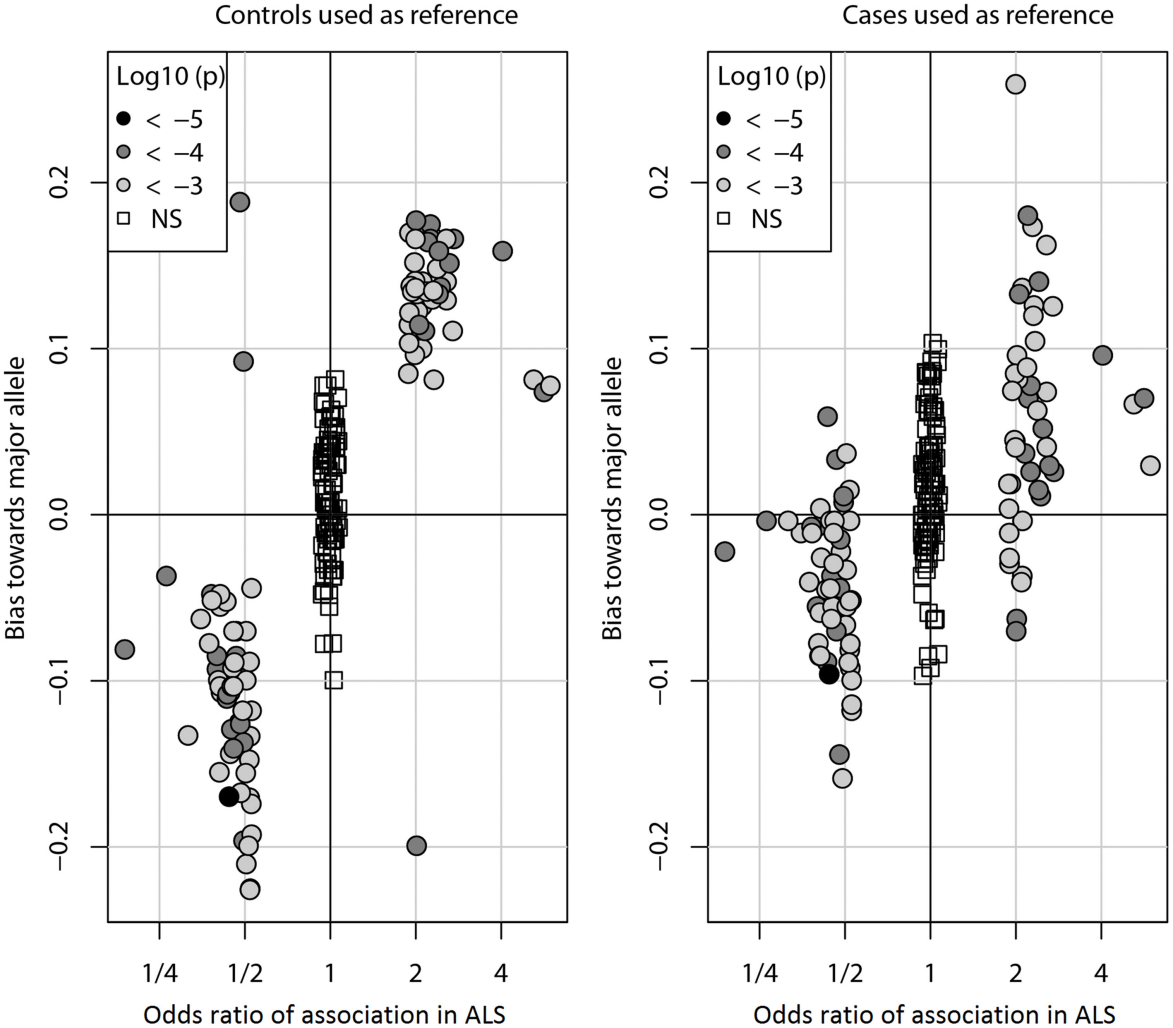
**Imputation bias vs. odds ratio of association in ALS**. Each circle represents one of the 100 DAM in ALS. For each SNP, the odds ratio (OR) of association (x-axis) indicates whether the minor allele (OR > 1) or the major allele (OR < 1) is the susceptibility allele (the allele more prevalent in cases than controls). The imputation bias (y-axis) indicates whether imputation error favors the major allele (positive values) or the minor allele (negative values). When controls were used as the reference for imputation, imputation is biased against the susceptibility allele. When an independent set of cases was used as the reference for imputation, the bias is significantly decreased. For reference, the 100 NAM (OR ≈ 1) are shown as boxes. Points are shaded by the log10 *p*-value of association with disease. The odds ratios of NAM are exaggerated for visual clarity.

Figure 1 shows the bias (Y-axis) when controls (left) or cases (right) are used as a reference to impute SNPs in the ALS dataset. With either reference population the bias is consistently against the risk allele and can be observed for all DAM (circles) including the most significantly associated SNPs (dark gray) as well as for more modestly associated (light gray). However, the magnitude of the bias is lower when cases are used as reference. We observed similar results in the PD and CD data sets (Supplementary Figures S1, S2). Of note, results were largely unchanged when the call tolerance parameter *T* was changed from 0.5 to 0.3 or 0.1 (data not shown), or when fractional genotypes were used (Supplementary Figures S3–S5).

**Figure 2:**
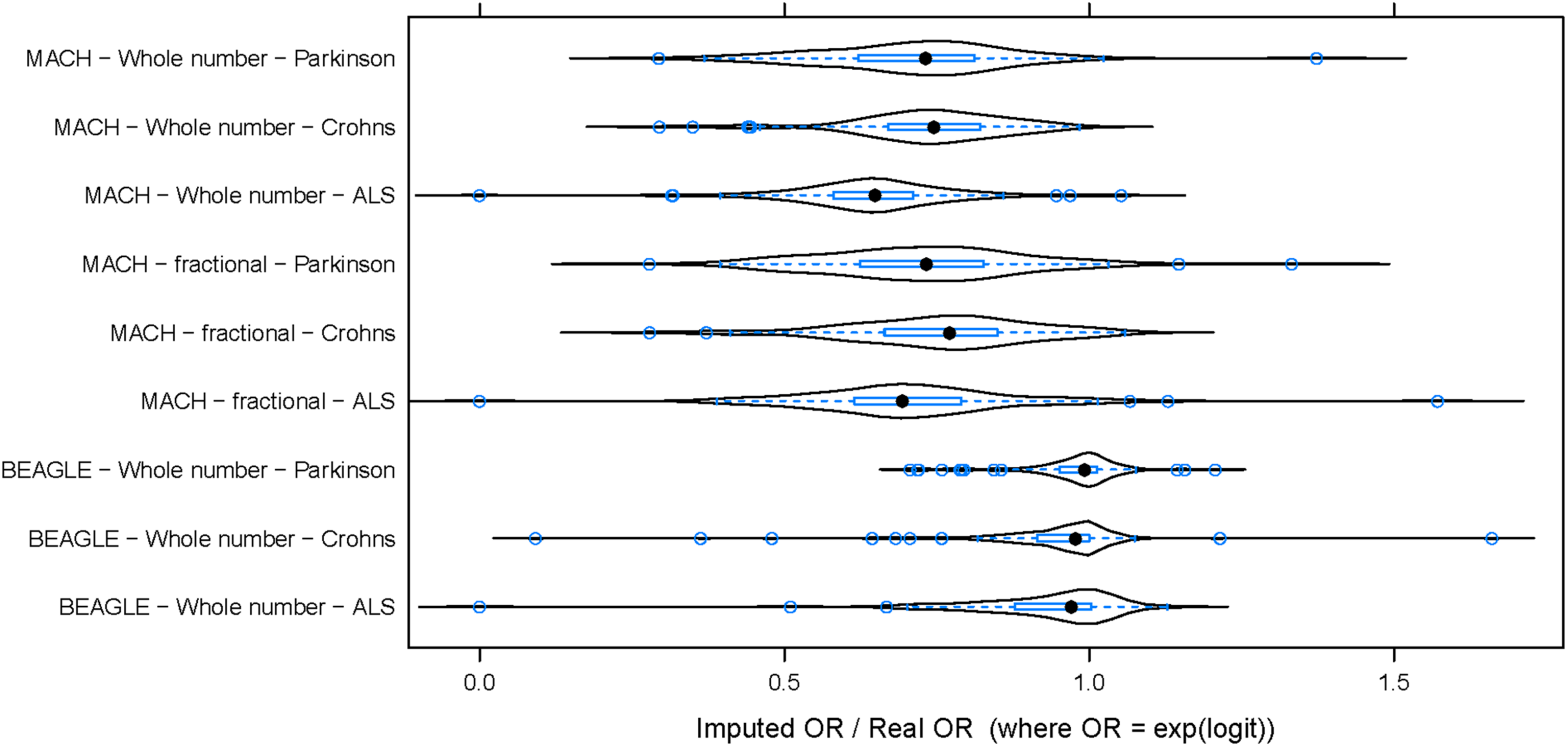
**The distribution of (imputed OR/true OR) for 100 DAM in each dataset**. In each of three datasets, 100 DAM were selected and the odds ratio of association with disease was estimated using both genotyped (true) data and imputed data. The ratio of imputed odds ratio to true odds ratio (x-axis) takes a similar distribution across the 100 SNPs in each disease. The odds ratio (OR) of association was generally lower for imputed data than for true data (imputed OR/true OR < 1). This hold true whether we use whole number imputation by Mach **(top)**, fractional imputation by Mach **(middle)**, or whole number imputation by Beagle **(bottom)**. In contrast to Mach imputation, the magnitude of the mean imputed OR was closer to the magnitude of the true OR for all three datasets when using Beagle imputation. ALS, Amyotrophic Lateral Sclerosis; PD, Parkinson's Disease; CD, Crohn's Disease.

To evaluate the potential effect of imputation on a genome-wide association study (GWAS), the same three datasets (PD, ALS, and CD) were used to perform three parallel association studies. For this analysis, imputation was performed using the CEU subset of the 1000 Genomes as a reference for imputation (Abecasis et al., 2010). For any SNP, the odds ratio (OR) was defined by logistic regression (OR = exp(*b*), where *b* is the estimate of the logit coefficient in the logistic regression). Odds ratios computed from imputed genotypes (imputed OR) were compared to those obtained using data from experimentally determined genotypes (from SNP chip; termed “true” OR). Imputed OR were compared to true OR for each DAM. Figure 2 shows the distribution of imputed/true OR for the top 100 DAM in each dataset. Using integer imputation, the magnitude of the mean imputed OR was only 64.2% ± 2.8 (mean ± 2 × standard error) as high as the magnitude of the true OR in ALS, 71.1% ± 3.2 in PD, and 73.8% ± 2.4 in CD. In all cases, it is evident that the distribution of OR is shifted toward the left of 1.0, a vertical dotted line which represents equality between imputed OR and true OR. Using fractional genotypes yielded similar results (Figure 2). Using another commonly used imputation software algorithm (Beagle) yielded similar results (Figure 2). However, in contrast to Mach imputation, the magnitude of the mean imputed OR was closer to the magnitude of the true OR in each dataset. In summary, the observed effect size after imputation is considerably lower for both types of imputation although in the second analysis using Beagle and a larger reference dataset improves the accuracy of imputation. It should be noted that for a few SNPs, the imputed OR is nearly equal or greater than the true OR (imputed OR/true OR ≥ 1). That is, for these SNPs, there is no decrease in observed magnitude of association with disease when imputed genotypes are used.

## Discussion

We have shown that imputation of DAM is consistently and significantly biased against the risk allele. This was seen in three diseases, and using two different software algorithms of imputation. The number of top SNPs selected as DAM or NAM was arbitrary and we acknowledge that many of these SNPs are likely to be false positives. However, since this arbitrary threshold was used for all datasets, we deemed this as a valid strategy. Furthermore, the fact that using independent cases as a reference (rather than controls) reduces the imputation bias indicates that at least some of these SNPs may be truly associated with disease.

While imputation bias is reduced when matching cases were used as reference for imputation, the effect is still present. We offer three possible explanations for the persistence of bias in this scenario. First, as in most common diseases, significant genetic heterogeneity could result in reference cases not carrying the same “risk” haplotype structure as the original cases, thus leading to underestimation of “risk” alleles during imputation. Second, the disease variants/mutations may occur in relatively small genetic windows which are not spanned by enough SNPs to make imputation effective. Third, the disease alleles are rare. If the frequency of the “non-risk” allele greatly outweighs the frequency of the “risk” allele, then the inherent bias for common alleles will add to the apparent bias for the “non-risk” allele. Future analyses aimed at describing the relative contribution of these hypotheses in diseases of varying genetic complexity are needed.

Although the Beagle imputed odds ratios are closer to the real odds ratios than the Mach imputed odds ratios, they are still significantly less than the real odds ratios. The reduced bias for Beagle imputation can be explained by at least two reasons. First, Beagle may be using a more accurate algorithm for imputation though this is unlikely given previously published head-to-head analysis of Beagle vs. Mach, and, second, the reference panel may be more up to date and consists of more individuals.

In conclusion, while combining datasets by imputation can lead to a more powerful GWAS (Becker et al., 2009; Hao et al., 2009) by allowing successful identification of SNPs associated with various phenotypes (Sanna et al., 2008; Willer et al., 2008; Zeggini et al., 2008; De Jager et al., 2009), the described decrease in signal inherent to imputation can partially offset any gain in power resulting from the combination of studies. Important implications of this finding include the fact that some truly associated variants may not be detected, and that some genome-wide significant findings may have larger true effect sizes than estimated. Since the imputation error of any given SNP cannot be known a-priori, individual genotyping of candidate SNPs by imputation should always be performed as a follow-up (Halperin and Stephan, 2009). In summary, imputation is a powerful method to estimate genetic risk at the population and individual level, but a careful choice of control population is required to minimize biases inherent to the approach. A plausible strategy is to consider deeper genotyping or whole genome sequencing of a small panel of ethnically matched cases and controls to be used as a reference for imputation.

## Methods

### Datasets: three case-control GWAS

Quality controlled, genotype-level data from three previously published independent case-control GWAS in individuals of European ancestry in Amyotrophic Lateral Sclerosis (ALS), Crohn's Disease (CD), and Parkinson's Disease (PD) were obtained from dbGAP (Mailman et al., 2007) (Supplementary Table S1). In CD (Rioux et al., 2007) and PD (Fung et al., 2006), cases and controls were matched by sex, age (or year of birth), and ancestry (Rioux et al., 2007; Simon-Sanchez et al., 2009). For ALS cases (Schymick et al., 2007), a sample from neurologically normal controls (Simon-Sanchez et al., 2007) were matched for age and gender and ancestry (Schymick et al., 2007). After quality control done by the original authors, we performed a second layer of quality control on markers (MAF > 0.05, HWE *p*-value > 10^−6^ in controls, genotype success rate > 95%).

For each disease dataset, half of the patients and a matched number of controls were extracted (split “A”) while maintaining original ratios of gender and age. This half of the data (split A) was used to perform a association study (using software Plink Purcell et al., 2007), using a genotypic model or a linear dose model. Here we report results using the genotypic model; results for the linear trend model were largely similar and not shown. The 100 markers with the largest absolute effect size (DAM) were considered for imputation (OR > 1.88 in ALS, OR > 1.53 in PD, OR > 1.44 in CD). As a control, 100 SNP markers with the smallest absolute effect size (NAM) were imputed with the same procedure (1 ≤ OR < 1.001).

For each marker of interest (DAM or NAM), genotypes of cases of split A were masked with the goal of predicting them using imputation. Next, imputation of the masked genotypes was carried out using all markers within a 1 Mb window centered on each query SNP. The imputation window size was chosen large enough so as to include neighborhood SNPs that have *r*^2^ > 0.2 with the query SNP. The imputation was repeated with each of two reference sets: (a) the previously unused healthy controls from split B of the same study, (b) an equal number of the previously unused affected case individuals from split B of the study.

Next, for each marker of interest (DAM or NAM) the genotypes of all cases and all controls from each study (split A and split B) were masked with the goal of predicting them with imputation. Imputation was performed using the CEU subset of the 1000 Genomes as a reference for imputation (Abecasis et al., 2010).

Imputation was carried out using Mach (Li et al., 2006) (see Nothnagel et al., 2009 for an evaluation of the relative performance of this algorithm compared to others), with default settings and default quality control criteria for SNPs (MAF > 0.05, HWE *p*-value > 10^−6^, genotype success rate > 95%). For each imputed genotype, Mach outputs a fractional genotype (*G*_imp_) between zero and two that corresponds to the inferred number of copies of the minor allele, where minor allele is determined using dataset frequencies (cases and controls). The fractional genotype is compared to the genotype by SNPchip (*G*_0_), or “true” genotype.

### Discordance and bias using integer genotypes

Using a call tolerance parameter (*T*), the integer imputed genotype (*G*_int_) is homozygous for the major allele if *G*_imp_ < *T*, heterozygous if 1 − *T* ≤ *G*_imp_ < 1 + *T*, or homozygous for the minor allele if *G*_imp_ ≥ 2 − T; otherwise the genotype is considered missing. Results using *T* = 0.5 are shown here. Results using stricter tolerance thresholds *T* = 0.3 and *T* = 0.1 were largely similar and are not shown. When using integer genotypes, discordance *D*_int_ = *D_M_* + *D_m_* is the percent of imputed genotypes that do not match genotypes from SNP chip, where *D_M_* is the percent of samples where rounded imputation mistakenly over-predicts the major allele in the genotype (by one or two copies), and *D_m_* is the percent of samples where imputation mistakenly over-predicts the minor allele in the genotype (by one or two copies). The bias *B* is defined as *B*_int_ = *D_M_* − *D_m_*. The bias *B*_int_ is positive when the major allele is over-predicted and negative when the minor allele is over-predicted. The bias cannot exceed the discordance: |*B*_int_| ≤ *D_int_*.

The discordance (or bias) calculated at individual SNPs is the sum (or difference) of two proportions. To compare groups of SNPs, a Gaussian approximation of the discordance (or bias) distribution was employed and *t*-statistics compared the difference in means between two groups of SNPs. Normality of the distributions were tested by the Anscombe–Glynn test of kurtosis. Further, non-parametric tests of differences in medians were used in parallel to confirm results.

### Discordance and bias using fractional genotypes

For each SNP, we computed across samples the mean discordance *D*_frac_ = mean |*G*_imp_ − *G*_0_|, and the mean bias *B*_frac_ = mean (*G*_imp_ − *G*_0_). The bias *B*_frac_ is positive when the major allele is over-predicted and negative when the minor allele is over-predicted across all samples. The bias cannot exceed the discordance: |*B*_frac_|≤*D*_frac_.

The discordance (or bias) calculated at individual markers is a mean across the samples. To compare groups of markers (e.g., DAM vs. NAM), a Gaussian approximation of the discordance (or bias) distribution was employed and *t*-statistics compared the difference in means between two groups of SNPs. Normality of the distributions were tested by the Anscombe–Glynn test of kurtosis. Further, non-parametric tests of differences in medians were used in parallel to confirm results (not shown).

## Author contributions

Sergio E. Baranzini, Pouya Khankhanian, and Pierre-Antoine Gourraud conceived and designed the study. Pouya Khankhanian and Lennox Din performed the data analysis. Stacy J. Caillier performed the assays. Sergio E. Baranzini, Pouya Khankhanian, Lennox Din, and Pierre-Antoine Gourraud wrote the paper. All authors read and approved the final manuscript.

### Conflict of interest statement

The authors declare that the research was conducted in the absence of any commercial or financial relationships that could be construed as a potential conflict of interest.

## Supplementary material

Supplementary Figure S1 **Bias vs. log odds ratio of association in Parkinson's Disease**. Analog of Figure 1 for Parkinson's Disease.

Supplementary Figure S2 **Bias vs. log odds ratio of association in Crohn's Disease**. Analog of Figure 1 for Crohn's Disease.

Supplementary Figure S3 **Analog of Figure 1 using fractional imputed genotypes**.

Supplementary Figure S4 **Analog of Figure S1 using fractional imputed genotypes**.

Supplementary Figure S5 **Analog of Figure S2 using fractional imputed genotypes**.
