# Supplementary figures and images for: SNP imputation bias reduces effect size determination
Source: Front Genet. 2015 Feb 9;6:30. doi: 10.3389/fgene.2015.00030 (PMC4321633; doi:10.3389/fgene.2015.00030)

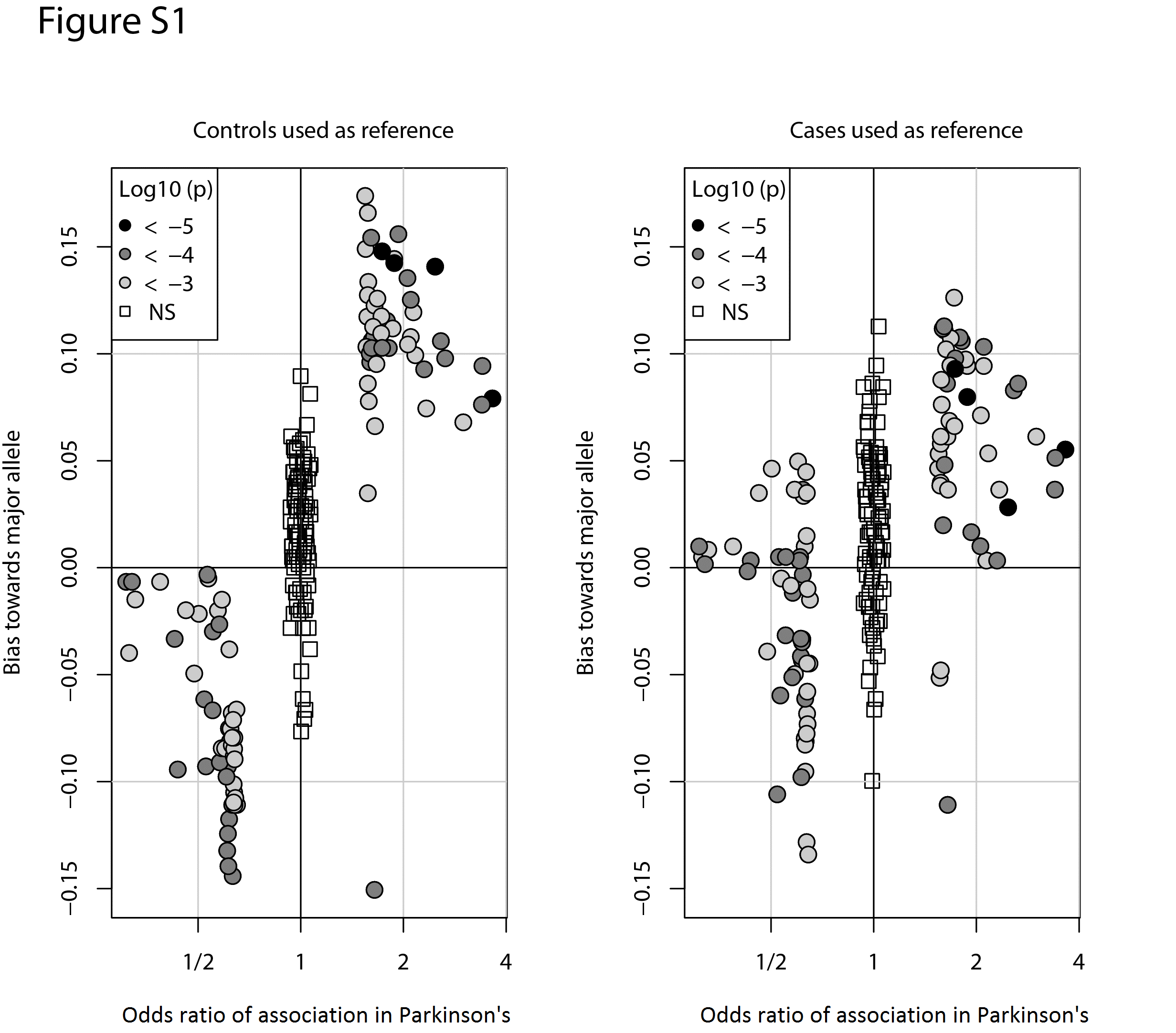

Supplement: Supplementary Figure S1 — Bias vs. log odds ratio of association in Parkinson's Disease. Analog of Figure 1 for Parkinson's Disease. [file Image1.TIF]

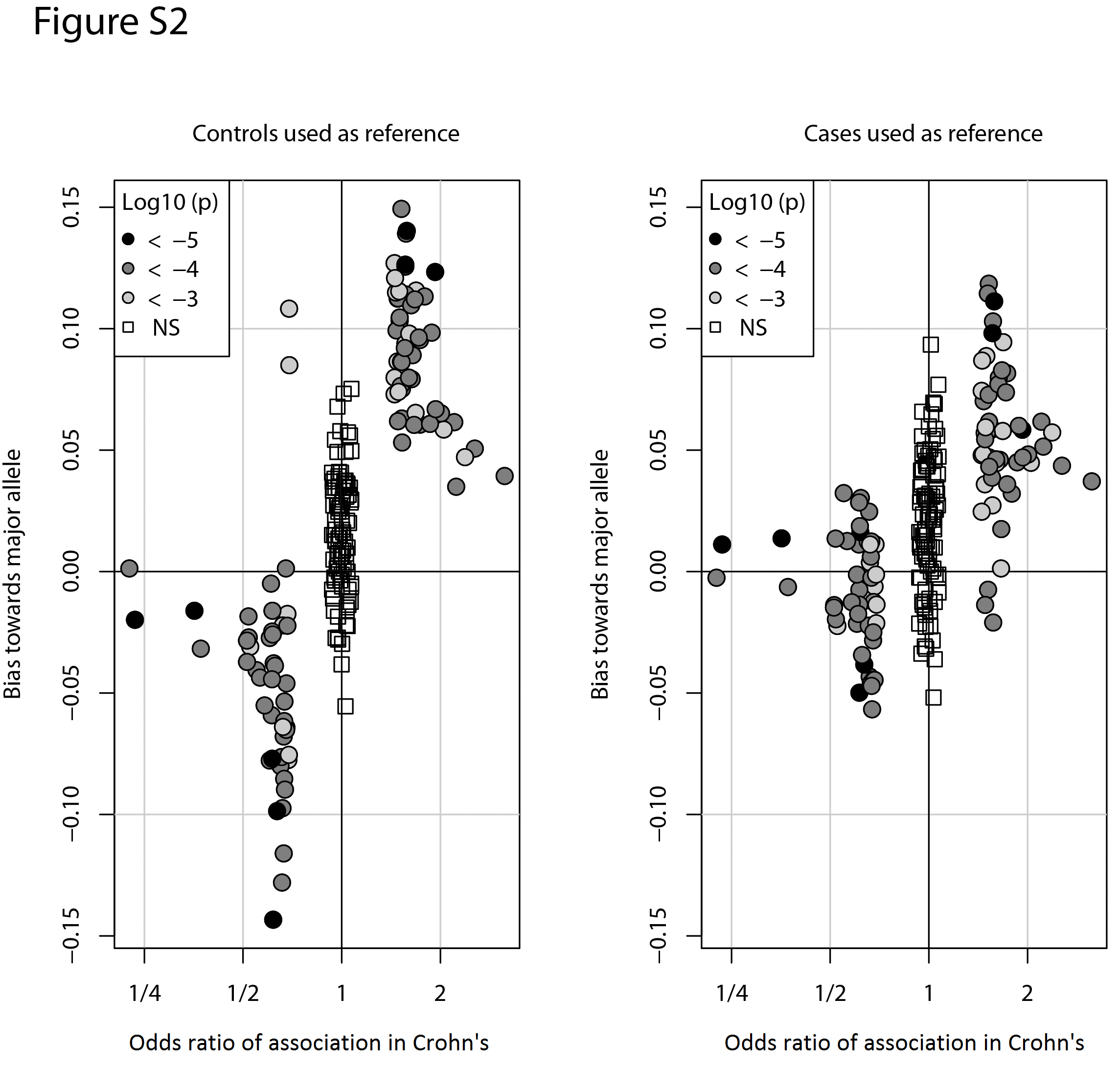

Supplement: Supplementary Figure S2 — Bias vs. log odds ratio of association in Crohn's Disease. Analog of Figure 1 for Crohn's Disease. [file Image2.TIF]

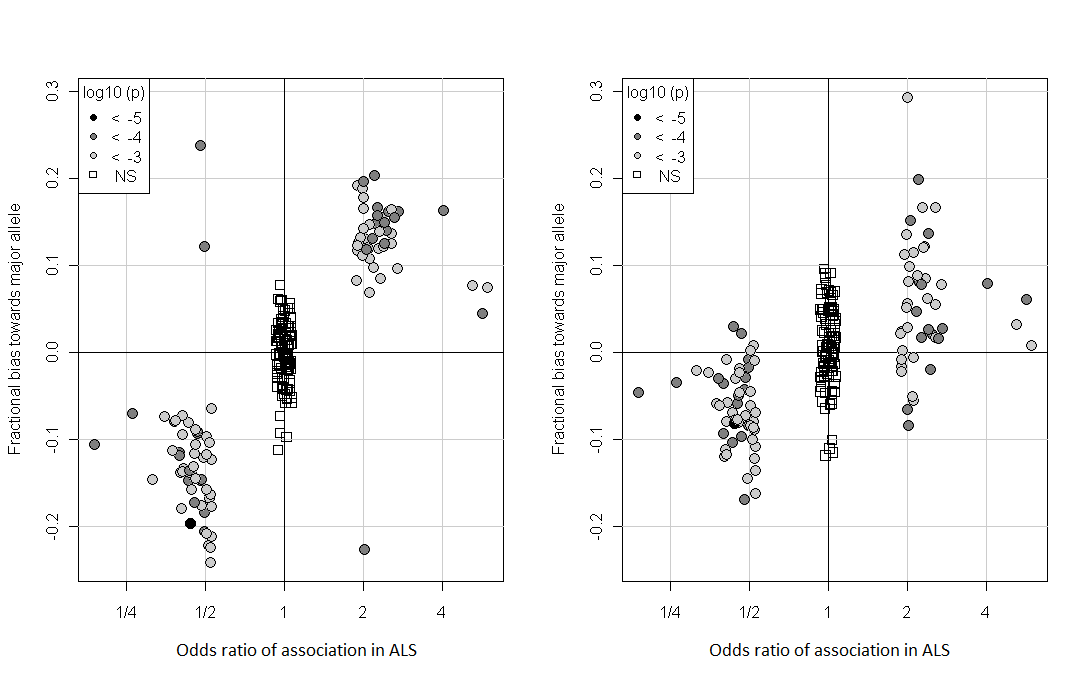

Supplement: Supplementary Figure S3 — Analog of Figure 1 using fractional imputed genotypes. [file Image3.TIFF]

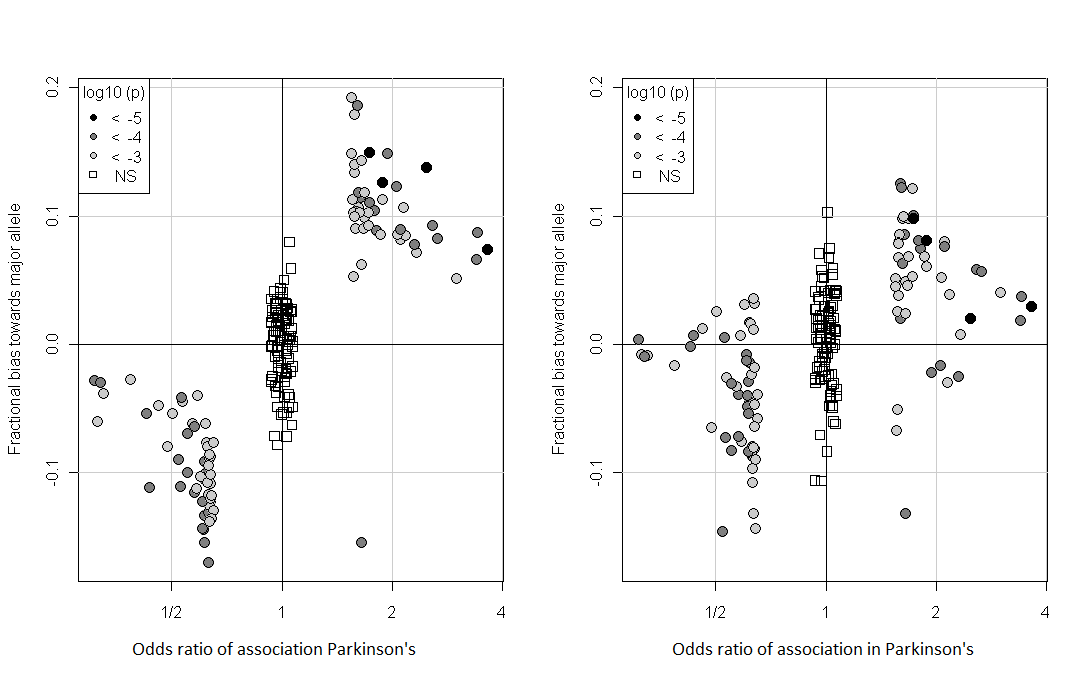

Supplement: Supplementary Figure S4 — Analog of Figure S1 using fractional imputed genotypes. [file Image4.TIFF]

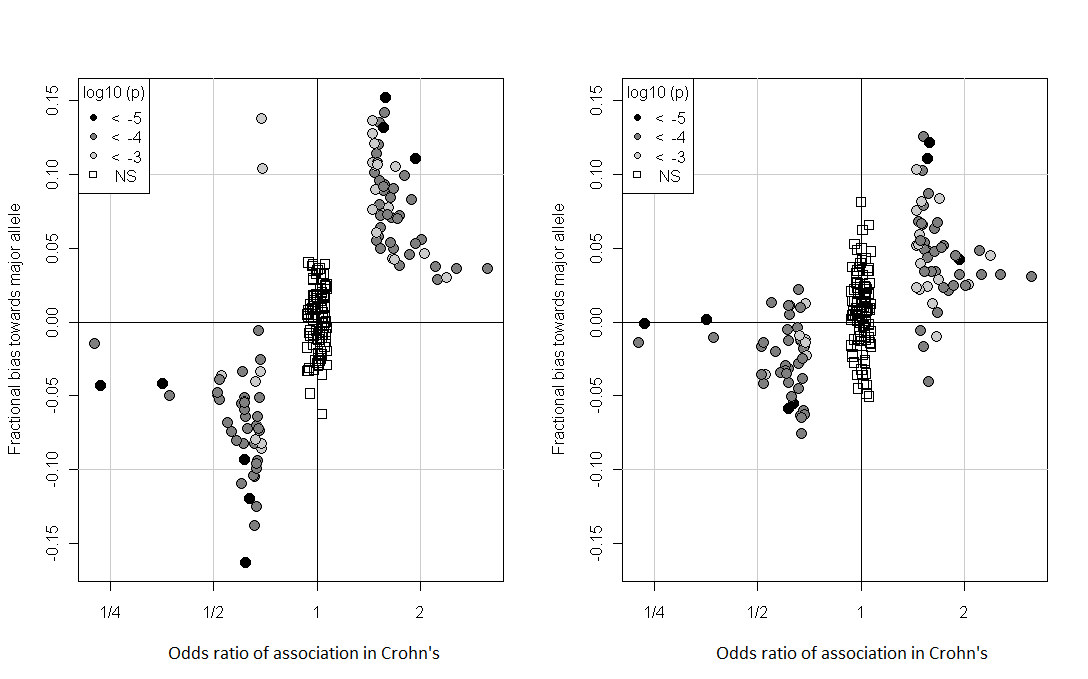

Supplement: Supplementary Figure S5 — Analog of Figure S2 using fractional imputed genotypes. [file Image5.TIFF]
