# Supplementary material for: SNP imputation bias reduces effect size determination
Source: Front Genet. 2015 Feb 9;6:30. doi: 10.3389/fgene.2015.00030 (PMC4321633; doi:10.3389/fgene.2015.00030)
Supplement: Supplementary file 6 [file DataSheet1.DOCX]

## Supplementary Table S1: Datasets

| Disease | Cases | Controls | Markers passing QC | Platform |
| --- | --- | --- | --- | --- |
| ALS | 276 | 266 | 307,124 | Illumina HumanHap300 |
| Parkinson's | 671 | 535 | 534,184 | Illumina HumanHap550 |
| Crohn's | 813 | 947 | 300,359 | Illumina HumanHap300 |

Data obtained from dbGAP include two complete case-control datasets: Parkinson's disease (phs000089) and Crohn's disease (phs000130). A third case-control dataset was created using 276 ALS cases (phs000101) and 266 neurologically normal controls (phs000004).
